# Using the Simulated Patient Methodology to Assess Paracetamol-Related Counselling for Headache

**DOI:** 10.1371/journal.pone.0052510

**Published:** 2012-12-27

**Authors:** Nejc Horvat, Marko Koder, Mitja Kos

**Affiliations:** Chair of Social Pharmacy, University of Ljubljana- Faculty of Pharmacy, Ljubljana, Slovenia; University of Würzburg, Germany

## Abstract

**Objectives:**

Firstly, to assess paracetamol-related counselling. Secondly, to evaluate the patient’s approach as a determinant of counselling and to test the acceptability of the simulated patient method in Slovenian pharmacies.

**Methods:**

The simulated patient methodology was used in 17 community pharmacies. Three scenarios related to self-medication for headaches were developed and used in all participating pharmacies. Two scenarios were direct product requests: scenario 1: a patient with an uncomplicated short-term headache; scenario 2: a patient with a severe, long-duration headache who takes paracetamol for too long and concurrently drinks alcohol. Scenario 3 was a symptom-based request: a patient asking for medicine for a headache. Pharmacy visits were audio recorded and scored according to predetermined criteria arranged in two categories: counselling content and manner of counselling. The acceptability of the methodology used was evaluated by surveying the participating pharmacists.

**Results:**

The symptom-based request was scored significantly better (a mean 2.17 out of a possible 4 points) than the direct product requests (means of 1.64 and 0.67 out of a possible 4 points for scenario 1 and 2, respectively). The most common information provided was dosage and adverse effects. Only the symptom-based request stimulated spontaneous counselling. No statistically significant differences in the duration of the consultation between the scenarios were found. There were also no significant differences in the quality of counselling between the Masters of Pharmacy and Pharmacy Technicians. The acceptability of the SP method was not as high as in other countries.

**Conclusion:**

The assessment of paracetamol-related counselling demonstrates room for practice improvement.

## Introduction

There is a worldwide increase in the number of medicines available and sold in pharmacies without a prescription [1]. Among other reasons, this process is caused by the global trend of down-scheduling prescription medicines to non-prescription status and shifts in patient preferences towards self-care and self-responsibility for health [2]. Over-the-counter (OTC) medications are increasingly available in retail stores and online pharmacies where pharmacists are not necessarily available for consultation [3], [4]. The lack of counselling coupled with easy access to OTC medications can mislead patients into regarding OTC medication as safe [3], [5]. However, studies demonstrate that the practice of self-medication presents a possible risk of abuse and the inappropriate use of medicines, which increases the incidence of drug-related problems and may compromise patient safety [6], [7].

Paracetamol is a clear example of the process described above. In the United Kingdom (UK), United States of America and Ireland, the greater availability of paracetamol in retail stores caused increased consumption, thus increasing the number of cases with paracetamol overdose [8], [9], [10]. A study in Ireland determined that two-thirds of the study participants who were hospitalised due to paracetamol overdose obtained paracetamol in supermarkets, local shops and gas stations [9]. In the UK, about half of all overdoses involved paracetamol or paracetamol-containing drugs [8]. Paracetamol also caused one-half of all cases of acute liver failure in the UK [11]. Additionally, chronic alcohol consumption increased its hepatotoxicity [12]. In Slovenia, paracetamol was the 12^th^ most commonly ingested drug by poisoned adult patients, reporting 48 cases of hospitalization between 2001 and 2005 [13].

Such alarming data has triggered many initiatives to change the legal status of paracetamol from the general sales list to prescription-only or at least a pharmacy-only medicine [8]. In the UK, paracetamol is still available on the General Sales List although some restrictions were introduced [8]. In Slovenia, paracetamol remains a pharmacy-only medicine despite demands to deregulate its sale [14].

Community pharmacies are potential sites where the risks involved with self-medication could be prevented. They have an overview of the prescription and OTC medications that patients are taking. Community pharmacists possess a high level of knowledge and are easily available to patients. This places them in a unique position to support self-medication. The exchange of drug-related information between the pharmacist and the patient is therefore considered critical to ensuring positive patient outcomes. [2], [15].

There have been several studies that attempted to evaluate patient counselling. Most commonly, these studies used surveys, diaries or observations as the data collection method. The latter seems to be the most reliable survey method in terms of accuracy, consistency and cost effectiveness [16], [17]. Observations can be either participant or non-participant. Non-participant observations, where the interactions between pharmacist and patient are observed by a trained researcher, can be a subject to the Hawthorne effect (a change of behaviour due to being observed) [18]. In this respect, the simulated patient (SP) method has proved a useful and objective tool for evaluating professional performance. [17], [19] A SP is an individual who has been trained to make a covert visit to a pharmacy in order to enact a scenario that will test a specific behaviour of a member of pharmacy staff, without the staff being aware of the SP’s identity or that they are being tested [1], [20]. The method focuses on actual behaviours rather than proxy measures [20]. The feedback of the SP, who is trained to be observant, is more reliable than that solicited from regular customers. Potential disadvantages include negative attitudes of pharmacists towards these covert visits and generalisability of the findings to other health problems [20], [21].

The primary aim of the study was to assess paracetamol related counselling using simulated patients. The secondary objectives were to evaluate the patient’s approach (symptom-based vs. direct product requests) as a determinant of counselling and to test the acceptability of the simulated patient method in Slovenian pharmacies.

## Methods

Paracetamol-related counselling was assessed using the simulated patients method. The Faculty of Pharmacy and the “Mariborske lekarne” Public Institute, which consists of 17 community pharmacies in the Maribor region, signed an agreement to cooperate in the study. Further to the agreement with the management, employees were given the opportunity to refuse participation after they were informed about the objectives and design of the study. The employees could refuse their participation at any time. In order not to jeopardize the results, a 3-month period was given as the time in which a simulated patient would visit the pharmacy. Participants were assured that the data gathered would be kept anonymous and strictly confidential. The details of the scenarios and the identity of the simulated patients were not disclosed. Thus, the study was conducted covertly in order to provide more reliable results [1]. An application for ethical review was sent to the National medical ethics committee, who decided that ethical approval was not required.

### Scenarios

Three different scenarios were developed: two of them were direct product requests (the patient asking for a specific brand of medicine containing paracetamol) and one was a symptom-based request (the patient asking for a medicine for a headache). All three scenarios dealt with the self-medication of a headache. The details of the scenarios are given in Table 1. The desirable outcome in scenarios 1 and 3 was the supply of an analgesic. In scenario 2, the pharmacist should refer the patient to a physician without dispensing any analgesic as it was a severe, long-duration headache experienced for the first time. In addition, the patient had been taking the paracetamol-containing medicine for too long and was concurrently drinking alcohol.

**Table 1:** Scenarios.

Scenario 1 (short-duration headache, direct product request)	
The patient enters a pharmacy and requests one pack of Lekadol*. If the pharmacist offers a comparable medicine (e.g. Panadol*, Daleron* or Maridol* ^)^,the simulated patient insists on receiving Lekadol.	
The pharmacist is given the following informationwhen asked:	The product is for the patient him/herself.
	The patient has had a headache for the whole day.
	The pain is described as mild, dull, low intensity, affecting both sides of the head.
	Headaches are not experienced often.
	There are no special factors that trigger/worsen the headache: the patient does not drink coffee, does not smoke, occasionally (once per week) drinks a glass of wine, is currently not under stress.
	The headache usually resolves in a day or two.
	In the past, Lekadol has proven efficient in similar cases; he/she was taking it for a day or two.
	The patient is familiar with the medicine.
	The patient does not take any other medicines.
	The patient does not experience any other medical conditions.
**Scenario 2 (long-duration headache, direct product request)**	
The patient enters a pharmacy and requests one pack of Lekadol*. If the pharmacist offers a comparable medicine (e.g. Panadol*, Daleron* or Maridol* ^)^,the simulated patient insists on receiving Lekadol.	
The pharmacist is given the following informationwhen asked:	The product is for the patient him/herself.
	The headache has lasted for more than 14 days.
	The pain is deep and often severe.
	A headache of this intensity and duration has not occurred before.
	There are no special trigger factors: the patient drinks 1–2 glasses of wine daily, does not smoke or drink coffee.
	Occasionally the headache gets milder, especially after taking medications.
	The patient has been taking Lekadol for 14 days now (is familiar with the medicine). The patient also takes other analgesics (Aspirin and Ketonal** in cases of severe pain). The patient takes 3 tablets of Lekadol or Aspirin daily.
	The patient does not experience any other medical conditions.
**Scenario 3 (short-duration headache, symptom-based request)**	
The patient enters a pharmacy and asks for medicine for headache.	
The pharmacist is given the following informationwhen asked:	The product is for the patient him/herself.
	The patient has had a headache for a day.
	The pain is described as mild, dull, low intensity, affecting both sides of the head.
	Headaches are not experienced often.
	There are no special factors that trigger/worsen the headache: the patient does not drink coffee, does not smoke, occasionally (once per week) drinks a glass of wine, is currently not under stress.
	The headache usually resolves in a day or two.
	In the past, Lekadol has proven efficient in similar cases.
	The patient does not take any other medicines.
	The patient does not experience any other medical conditions.
**If the pharmacist does not ask any questions (except about familiarity with the medicine), the patient enquires whether there is anything** **he/she should pay attention to (valid for all three scenarios).**	

* Trade names of paracetamol-containing medications.

** Trade name of ketoprofen-containing medication.

A headache was selected because it is one of the most common health problems for which patients visit community pharmacy. Its lifetime prevalence is more than 90% [22]. Furthermore, the Slovenian Chambers of Pharmacy published a counselling protocol for headaches that was supposed to be applied in Slovenian pharmacies [23]. This protocol was the basis for the design of the scenarios and evaluation criteria.

### Simulated Patients

Three simulated patients (two females and one male) of varying ages were selected from the researchers’ acquaintances. The personal characteristics sought were reliability, intelligence and the ability to improvise. In order to minimise the possible detection of the simulated patients due to a different accent, local residents were chosen. The researchers delivered a one-day training course designed to help the simulated patients (SPs) play their roles. During the training, they were informed about the method, scenarios and rules of approach. A considerable part of the training involved role playing where one of the researchers acted as a pharmacist. The SPs played all three scenarios, which were also audio recorded. Further instructions and advice were given on the basis of the simulated patients’ performance. The SPs signed a contract with the Faculty in which they agreed to conform to study protocols. They also consented to the ethical code designed for this study in order to maintain the anonymity of participating individuals and protect the integrity of the data obtained.

### Pilot Study

A pilot study was performed to detect possible shortcomings of the methodology. The chosen pharmacy that agreed to participate was informed about the methodology, the aim of the pilot study and the time period in which the visits would take place. The SPs visited the participating pharmacy using various scenarios. All three scenarios and all three SPs were tested. In an interview that followed the last SP visit, the participating pharmacists stated that they did not detect any of the SP visits. No significant deficiencies in the constructed methodology were identified.

### Study Conduct

All pharmacy visits were audio recorded in order to avoid relying on the human cognitive processes, which is seen as a potential weakness of the simulated patient method [16].

During the study, every pharmacy was scheduled to receive three visits from the simulated patients. No simulated patient was scheduled to visit the same pharmacy more than once and no pharmacy was scheduled to receive the same scenario more than once. The study duration was 3 weeks.

After entering the participating pharmacies, the simulated patients requested either the purchase of a non-prescription medicine or treatment for a symptom according to the scenario used. The SPs were instructed to convey their request in a standardized way. Other details and information on the scenario were not given to the counsellor unless asked. During the visits, the SPs also tried to identify the counsellor’s profession (i.e. Master of Pharmacy or Pharmacy Technician) using either received bills or name tags.

### Visit Evaluation

Immediately after each visit, the SPs documented the counselling process on the evaluation form. The assessment was performed outside the pharmacy. Evaluation criteria were set for each of the scenarios on the basis of the published protocol of counselling in the case of a headache [23]. The criteria were divided into two main categories with further subcategories:

- Counselling content
  - information request (questions pharmacists should ask the patients),
  - information provision (information on medicine that pharmacists should give to the patients).
- Manner of counselling
  - the accuracy of pharmacist’s decision (dispensing the medicine/advice to visit a physician),
  - counselling spontaneity (spontaneously, counselling on request, no counselling),
  - counselling comprehensiveness (not comprehensible, almost not comprehensible, fairly comprehensible, mostly comprehensible, completely comprehensible).

Most items were designed to enable a yes/no scale for assessment. Since the pharmacist’s accurate decision in scenario 2 was not to dispense an analgesic, the scoring of the information provision subcategory differed from Scenarios 1 and 3 (details of the scoring system are in Table 2). Counselling spontaneity and counselling comprehensiveness were assessed on a grading scale. Each visit was scored according to a predetermined scoring system. The two categories were balanced to contribute 50% to the total score. The maximum total score for a single visit was 4 points. The composite score mainly served for comparisons (scenarios, profession, evaluators).

**Table 2:** The Scoring System for all Three Scenarios.

Category	Subcategories	Items	Maximum score forsubcategory	Weight
COUNSELLING CONTENT	questions pharmacists shouldask patients	Who is the product for/who has the headache?	**1 point**(each question is0.11 of the point)	**1**
		When has the headache occurred/how longhas the headache been present/howoften does it occur?		
		Which area of the head hurts?		
		Are there any other symptoms?		
		How intense is the pain/has pain of this intensityoccurred before?		
		What triggers/worsens the headache (e.g. coffee,smoking, alcohol)?		
		When does the pain get better?		
		Have you tried anything already/does it help/howlong have you been taking it?		
		Are you taking any other medication (purchasedor prescribed)?		
	information about the medicinethat pharmacists should giveto the patients	Scenarios 1 and 3: each item is 0.2 of the point:dosage, interactions, adverse effects, hepatotoxicity,written instructions	**1 point**	**1**
		Scenario 2: each item is 0.5 of the point: interactionwith alcohol/hepatotoxicity, taking paracetamolfor too long		
MANNER OF COUNSELLING	The accuracy of the pharmacists’decision (dispensing the medicine/advice to visit a physician)	Scenarios 1 and 3: dispensing the medicine	**1 point**	**0.67**
		Scenario 2: advice to visit a physician		
	counselling spontaneity	spontaneously = 1	**1 point**	**0.67**
		on request = 0.5		
		no counselling = 0		
	counselling comprehensiveness	completely comprehensible = 1	**1 point**	**0.67**
		mostly comprehensible = 0.75		
		fairly comprehensible = 0.5		
		almost not comprehensible = 0.25		
		not comprehensible = 0		
TOTAL				**4**

After all the visits were completed, reports and audio recordings were collected. Audio recordings were transcribed using the Nvivo v8, a software programme for the management and analysis of qualitative data [24]. The transcribed interactions were assessed independently by two researchers who later agreed upon the final assessment. Thus, each visit was evaluated by the SP and the researchers. In this way, the need for audio recording in future studies could be established. Both SPs and the researchers were independent of the participating pharmacies, thus reducing the potential bias.

Visits in which one of the following conditions was fulfilled were excluded from further evaluation:

- the dispensed medicine did not contain paracetamol,
- the pharmacist detected the SP visit,
- the SP did not follow the scenario.

The study analysis was based on the researchers’ assessment of the pharmacy visits since more valid results were expected. The audio recordings also served to define the duration of consultation in each encounter.

### Detection of the Simulated Patient Visits

It was important that the simulated patients were not detected by the participating pharmacists since their behaviour may have changed if they suspected a covert visit. In order to determine whether the pharmacy staff detected the simulated patients’ visits, a special form was prepared and sent to the participating pharmacies. The pharmacists were asked to complete the form with details of all suspected visits. On the basis of the information provided, the researchers would be able to determine if a visit had been detected and eliminate it from further study.

### Feedback from Pharmacies

On completion of the study, an online questionnaire was created and notification sent to all the participating pharmacies. The aim was to determine the pharmacists’ opinions regarding the acceptability of the methodology and their expectations for future studies. The questionnaire included six statements that were evaluated on a scale from 1 to 5 (''Fully disagree'' to ''Fully agree''):

1. The evaluation of pharmacy services using the simulated patient method is intended solely for the management’s inspection of pharmacy employees.
2. The evaluation of pharmacy services using the simulated patient method is controversial and should therefore not be performed in pharmacies.
3. The evaluation of pharmacy services using the simulated patient method is sensible, since pharmacy service quality can be improved.
4. The announcement of the on-going evaluation of pharmacy services has automatically increased the quality of work in our pharmacy.
5. The simulated patient method should be used when introducing new pharmacy services or changing existing ones.
6. In the future, the majority of our employees would be willing to participate in research studies using the simulated patient method.

An e-mail was sent to all the participating pharmacies in which they were asked to discuss these statements among the pharmacy employees and then evaluate them.

### Statistical Analysis

The collected data was analysed using the statistical software SPSS v17 [25]. Each visit to every pharmacy was evaluated in accordance with a predefined scoring system to obtain a score between 0 and 4. Initially, the descriptive statistics was completed. The counselling content, spontaneity and comprehensiveness were determined. Afterwards, the obtained scores of all the visits were examined for distribution normality. Since the Kolmogorov-Smirnov test did not show normal distribution, the non-parametrical tests (Wilcoxon rank sum, Wilcoxon signed rank or Kruskal-Wallis test) were used in the further analysis. The differences in counselling score and the duration of the consultation between the direct product request and the symptom-based request were investigated. Next, the visit evaluation scores from the SPs and the researchers were compared. Finally, the effect of the profession on the counselling score was determined.

## Results

All 17 pharmacies participated in the current study. Since three visits were made per pharmacy, 51 evaluation forms and audio recordings were received from the simulated patients. All the evaluation forms were filled in appropriately and none contained any exclusion criteria. The audio recordings were comprehensible. The participating pharmacists did not report suspecting any of the simulated patients’ visits. The pharmacies were given feedback regarding their performance.

### Counselling Content

The first category in the evaluation criteria was the counselling content, where the pharmacists’ information request and provision was assessed. In the case of scenario 1 (short-duration headache, direct product request), the pharmacists rarely asked the simulated patients any questions. In just two out of the seventeen participating pharmacies, the simulated patients were asked for whom the product was for. However, information provision was more common. The most frequently provided information was the dosage and adverse effects. The results of scenario 2 (long-duration headache, direct product request) were similar. The most commonly asked question was who the product was for (5 out of 17 pharmacies). Information on the interactions of paracetamol with alcohol and/or its hepatotoxicity was given in 4 pharmacies. In scenario 3 (short-duration headache, symptom-based request), asking questions was more common: the majority of pharmacists asked who had the headache (14 out of 17) and whether the patient has tried anything already (12 out of 17). In 9 pharmacies (53%), information about dosage and adverse effects were provided. Details of the counselling content are listed in Table 3.

**Table 3:** Counselling Content.

		Scenarios*		
Subcategories	Items	Scenario 1	Scenario 2	Scenario 3
Information request	Who is product for/who has the headache?	2 (12%)	5 (29%)	14 (82%)
	When has the headache occurred/how long has theheadache been present/how often does it occur?	1 (6%)	0	0
	Which area of the head hurts?	0	0	0
	Are there any other symptoms?	0	1 (6%)	0
	How intense is the pain/has pain of this intensityoccurred before?	0	0	7 (41%)
	What triggers/worsens the headache (e.g. coffee,smoking, alcohol)?	0	0	0
	When does the pain get better?	0	0	0
	Have you tried anything already/does it help/how long haveyou been taking it?	0	0	12 (71%)
	Are you taking any other medication(purchased or prescribed)?	0	0	0
Information provision	Dosage	12 (71%)	n/a	9 (53%)
	Interactions	2 (12%)		1 (6%)
	Adverse effects	8 (47%)		9 (53%)
	Hepatotoxicity	7 (41%)		2 (12%)
	Written instructions	1 (6%)		0
	Interaction with alcohol/hepatotoxicity	n/a	4 (24%)	n/a
	Taking paracetamol for too long		0	

* The numbers present the number of pharmacies in which specific information (items) was requested from the patients or conveyed to the patients. The numbers in brackets are percentages of all the participating pharmacies (17).

### Counselling Spontaneity

In the case of direct product requests (scenarios 1 and 2), no pharmacist counselled spontaneously. Most of them counselled at the request of the simulated patient and some did not counsel at all. On the other hand, in the case of a symptom-based request (scenario 3), all the counselling was spontaneous.

### Counselling Comprehensiveness

The simulated patients assessed the counselling comprehensiveness on a scale of 1 to 5. The mean score was 2.8±1.0. 23 out of 51 visits (45%) were considered not comprehensible or almost not comprehensible. If the counselling did not take place (in 3 cases), it was regarded as not comprehensible. The simulated patients considered 3 out of the 51 visits (5.9%) to be completely comprehensible.

### Profession as a Determinant of Counselling Quality

The counsellor’s profession was identified during the encounters using either the bills received or name tags. In most cases where the identification of profession was possible (42 out of 51 visits), Masters of Pharmacy performed the counselling. The comparison of counselling scores between the Masters of Pharmacy (N = 27) and the Pharmacy Technicians (N = 15) yielded no significant differences (Wilcoxon rank sum test: p = 0.823; α = 0.05).

### Direct-Product Request vs. Symptom-Based Request

The mean scores for each scenario were calculated using the researchers’ evaluations from all 17 participating pharmacies. The maximum total score was 4. The results are shown in Table 4.

**Table 4:** Comparison Between Scenarios.

		Scenarios		
		Scenario 1	Scenario 2	Scenario 3
Total score (max 4)	Simulated patients’ evaluation	1.38±0.41	0.61±0.37	1.86±0.53
	Researchers’ evaluation	1.64±0.50	0.67±0.38	2.17±0.39
Duration of consultation [s]		52.4±13.9	47.9±18.5	65.3±32.6

The mean scores differed significantly between scenarios (Kruskal-Wallis test: p<0.001; α = 0.05). The highest mean score was obtained in scenario 3 and the lowest in scenario 2. The lowest score in scenario 2 was also attributed to an inaccurate pharmacists’ decision to dispense an analgesic instead of referring the patient to a physician. When comparing scenarios 1 and 3 (which are essentially the same, the difference being the simulated patient’s approach: a direct product request vs. symptom-based request), a significant difference was found (Wilcoxon rank sum test: p = 0.007; α = 0.05). The counselling in the case of a symptom-based request was evaluated with substantially higher scores than in the case of direct product request.

Although the mean scores between the scenarios differed significantly, the duration of the consultation did not. Table 4 shows the mean duration of the consultation in seconds for each scenario. The consultation was the longest in the case of scenario 3 and the shortest in scenario 2. However, there were no statistically significant differences in the duration of consultation when comparing all the scenarios (Kruskal-Wallis test: p = 0.138; α = 0.05).

### Comparing Evaluations made by Simulated Patients and the Researchers

When investigating the differences between simulated patients’ and researchers’ visit evaluations, statistically significant differences in scenarios 1 and 3 were established (Wilcoxon signed rank test: p = 0.039 and 0.008, respectively; α = 0.05). In scenario 2, the evaluations were similar. In all cases, the researchers evaluated visits with higher scores than the simulated patients. The differences in visit evaluations highlight the need to audio-record these interactions.

### Feedback from the Pharmacies

**Figure 1:**
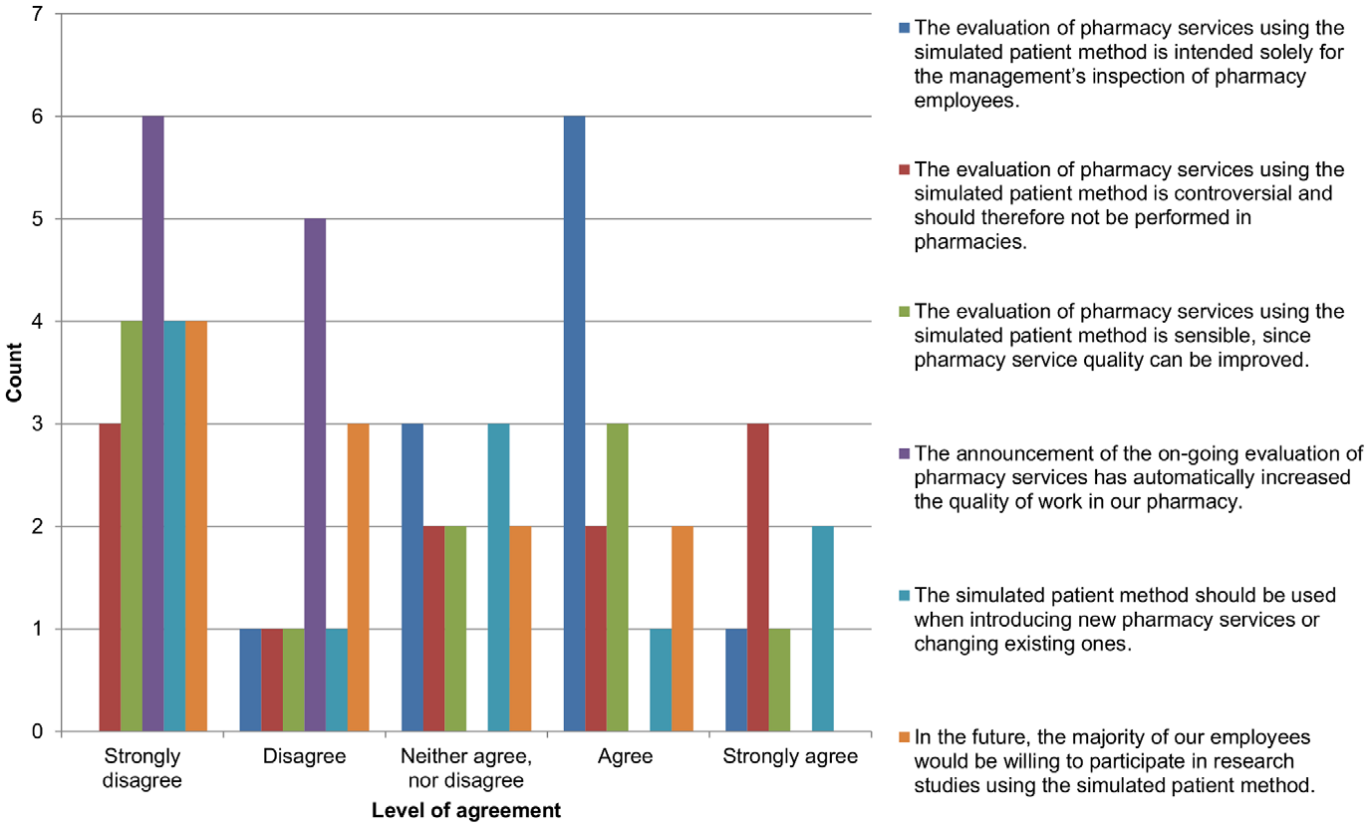
Feedback from Pharmacies. Participating pharmacists’ opinions regarding the acceptability of the simulated patient methodology and their expectations for future studies.

Feedback was received from 11 pharmacies (a 65% response rate). Their evaluation of the six statements is shown in Figure 1. The level of agreement with the statements is displayed as a bar chart. The ordinate axis presents the number of pharmacies that have chosen the corresponding option. The strongest agreement was found for statement 1 “The evaluation of pharmacy services using the simulated patient method is intended solely for the management’s inspection of pharmacy employees.” The highest level of disagreement was indicated for statement 4 “The announcement of the on-going evaluation of pharmacy services has automatically increased the quality of work in our pharmacy.” Four out of the 11 pharmacies that responded (36%) stated that they are not willing to participate in future research studies using the simulated patient method.

## Discussion

This study demonstrates pharmacy staff offered professional counselling, particularly in case of symptom-based requests (scenario 3). The counselling was spontaneous in all 17 participating pharmacies. Pharmacists requested and supplied more information than in the case of direct product requests. Giving information was the focus of counselling. The symptom-based scenario initiated more discussion and effort from the pharmacist as the patient needed to actually describe the symptoms instead of just asking for a specific product [6], [26]. Overall, the two most commonly provided items of information were dosage and adverse effects. Similar SP studies revealed that dosage counselling is one of the most frequently given instructions [27], [28], [29]. On the other hand, advice on adverse effects was given in half of all the cases in the current study, which is considerably more than in similar studies [6], [27].

This study also revealed substantial room for practice improvement. Taking into consideration negative experiences related to paracetamol hepatotoxicity and cases of overdose from abroad, it is imperative to achieve and maintain high quality counselling in pharmacies [8], [10], [11]. The lack of the latter is especially seen in direct product requests (scenarios 1 and 2), where counselling was not offered spontaneously and where the request for information was minimal. Furthermore, pharmacists did not detect that self-medication was inappropriate in scenario 2. The effect of the patient’s approach (symptom-based vs. direct product requests) as a determinant of counselling was in concordance with other studies [1], [6], [19]. The literature suggests three main reasons underlying this kind of behaviour [6], [7], [19], [26], [30]:

1. Lack of time and manpower.
2. Pharmacists focus on the product instead of the patient. The assessment of the patient’s information needs is thus based on their medicines instead of asking questions.
3. Pharmacists may assume that patients requesting a specific medicine have the knowledge to use it. Therefore pharmacists feel that asking questions and giving additional advice is patronizing and fear a negative patient reaction.

According to community pharmacists’ opinion, there is a shortage of practicing pharmacists, which could be addressed by a better system for the economic evaluation of pharmacy services. [31] Furthermore, mechanisms should be established to promote patient-centred care and to equip pharmacists with skills to effectively interact with patients [7], [20]. This would also improve counselling comprehensiveness. Furthermore, the risk of providing unwelcomed advice is likely to be much lower than the risk of drug-related problems without appropriate counselling [6].

### The Generalisability of the Findings

For the purpose of the study “Mariborske lekarne” Public Institute was selected. This is one of twenty four regional public institutes in Slovenia. The institute consists of 17 community pharmacies in the Maribor region and covers urban as well as rural area. The results are valid for the 17 community pharmacies included.

A random sample of all Slovenian pharmacies would be a more adequate approach in order to generalise results for Slovenia. However, the following reasons limited implementation of the simulated patient method on a national level. Firstly, most pharmacists in Slovenia have not been acquainted with this method. Therefore, provision of in-depth information was needed and agreements with officials of the pharmacies reached before starting the study. This was feasible for community pharmacies within one public institute. Secondly, pharmacists in some community pharmacies have a negative experience by the method as it was used as a kind of surveillance for commercially driven business, often neglecting the pharmacist role. Thirdly, ethical standards regarding the simulated patient method are not yet defined in Slovenia. Namely, the National medical ethics committee’s opinion was that ethical approval is not required for such studies. Moreover, other bodies that would evaluate ethics of the proposed study do not exist. Therefore, the researchers decided to implement the methodology in a selection of pharmacies and learn from the experience. At the same time the ethical code for such studies was built and the feedback from pharmacists included in the study collected.

Nevertheless, it would be hard to expect significantly different results in other Slovenian pharmacies. All pharmacists have undergone the same education program at the only Faculty of Pharmacy in the country and follow the same postgraduate courses primarily organized by the Faculty and the Slovenian Chamber of Pharmacies. The latter also provides guidelines and protocols for practice. Furthermore, the study was conducted in a public institute, which is a predominant organization of pharmacy practice in Slovenia (211 community pharmacies of 310 in the country). Public institutes, which are regionally organized, share a development and organization history.

### Counselling Protocol

In Slovenia, a protocol for headache counselling was published and disseminated in 2007 [23]. It lists a range of questions pharmacists should ask the patient when dealing with headaches. It also includes decisions on self-medication deriving from patient’s answers. The current study used this protocol to assess the counselling. To ensure they make an accurate decision in the current study, pharmacist would need to ask patients how long the headache has been present, whether they have taken anything already and how long they have been taking it. Consequently, the answers would prevent the pharmacists dispensing paracetamol in scenario 2. However, the applicability of the protocol in all situations is questionable. Some might argue that the counselling protocol content is too extensive, impractical and not necessary to follow in each situation. In this respect, it would be sensible to revise it from the communication and feasibility aspect, e.g. defining questions and information that are absolutely necessary in each situation, the pharmacist’s approach to the patient and so on.

### Profession as a Determinant of Counselling

Studies that applied the simulated patient method in the pharmacy setting have shown that the quality of counselling is not consistent at all times. It is influenced by a number of factors such as staff age and profession, prescription type (new or refill), drug class, pharmacy size, patient age and gender, the busyness and layout of the pharmacy, regulatory mechanisms etc. [7], [27], [28], [29] This study examined profession of pharmacists (Master of pharmacy, Pharmacy technician) as a determinant of counselling. No significant differences were found, which is in contrast to some other studies that established a connection between staff profession and consultation score [28], [29]. The small sample size may have caused this discrepancy.

### The Acceptability of the Simulated Patient Method

The acceptability of the SP method, measured by the questionnaire sent to all the participating pharmacies after the SP visit, was not as high as in similar studies [1], [6], [20]. The participants felt that the study is intended for management inspection and therefore deemed it controversial. These negative attitudes might have originated in the strictly commercially driven use of this method in some other Slovenian pharmacies in the recent past. As a result, the SP method has a negative connotation in Slovenian pharmacy practice.

Before using the simulated patient method in practice, it is essential to address the negative beliefs concerning the method. Participants should be informed about the aims of the SP method in greater detail. It should be explained that this method is a tool for improving the quality of pharmacy practice and not a way to sanction their performance. The conduct of the study should be highlighted and the consequences of the results clarified. Thus, in the longer term it is essential to establish positive experiences with the simulated patient method to increase its acceptability in practice.

### Conclusions

In summary, the assessment of paracetamol-related counselling in pharmacy displays room for improvement. Pharmacy patients who present their symptoms are offered more thorough counselling in terms of information request and information provision than patients who request a product. The acceptability of the simulated patient methodology used was not as high as in similar studies due to negative experiences with this method in the recent past.
